# *Arabidopsis* E3 Ubiquitin Ligases PUB22 and PUB23 Negatively Regulate Drought Tolerance by Targeting ABA Receptor PYL9 for Degradation

**DOI:** 10.3390/ijms18091841

**Published:** 2017-08-24

**Authors:** Jinfeng Zhao, Linlin Zhao, Ming Zhang, Syed Adeel Zafar, Jingjing Fang, Ming Li, Wenhui Zhang, Xueyong Li

**Affiliations:** 1 National Key Facility for Crop Gene Resources and Genetic Improvement, Institute of Crop Sciences, Chinese Academy of Agricultural Sciences, Beijing 100081, China; zhaojinfeng@caas.cn (J.Z.); 17745124980@163.com (M.Z.); adeelzafarpbg@gmail.com (S.A.Z.); fangjingjing@caas.cn (J.F.); 2 College of Life Sciences, Liaocheng University, Liaocheng 252059, China; zhaolin19871222@163.com (L.Z.); zhangwenhui0905@126.com (W.Z.); 3 College of Agriculture, Northeast Agricultural University, Harbin 150030, China; liming@neau.edu.cn; 4 Heilongjiang Academy of Agricultural Sciences, Industrial Crop Institute, Harbin 150086, China

**Keywords:** ABA receptor, cell-free degradation assay, degradation, drought tolerance, protein extract, U-box E3 Ubiquitin ligases

## Abstract

Drought causes osmotic stress and rapidly triggers abscisic acid (ABA) accumulation in plants. The roles of various ABA receptors in drought tolerance and molecular mechanisms regulating ABA receptor stability needs to be elucidated. Here, we report that Arabidopsis plants overexpressing PYL9, one of the 14 pyrabactin resistance (PYR)/pyrabactin resistance-like (PYL)/regulatory component of ABA receptors (RCAR) family ABA receptors, gained drought tolerance trait. Osmotic stress induced accumulation of the PYL9 protein, which was regulated by the 26S proteasome. PYL9 interacted with two highly homologous plant U-box E3 ubiquitin ligases PUB22 and PUB23. In the cell-free degradation assay, the degradation of GST-PYL9 was accelerated in protein extract from plants overexpressing PUB22 but slowed down in protein extract from the *pub22 pub23* double mutant. The in vivo decay of Myc-PYL9 was significantly reduced in the *pub22 pub23* double mutant as compared with the wild-type. Additionally, PUB22 also interacted with other ABA receptors such as PYL5, PYL7 and PYL8. Considering the improved drought tolerance in the *pub22 pub23* double mutant in previous studies, our results suggest that PUB22 and PUB23 negatively regulate drought tolerance in part by facilitating ABA receptors degradation.

## 1. Introduction

Phytohormone abscisic acid (ABA) plays crucial role in plant life cycle, including seed development, germination, dormancy, and response to abiotic stresses [1,2,3]. ABA was rapidly produced when plants suffered from abiotic stresses such as drought, salt or low temperature, which led to decreased water availability, namely osmotic stress [4,5]. There are ABA-dependent and ABA-independent signaling pathways in plants in response to osmotic stress [6,7]. ABA stimulates stomatal closure, regulates gene expression, and mediates physiological response to environmental changes in ABA-dependent signaling [5,8,9,10]. Proline and osmoregulatory solute usually accumulate in plant cells and maintain the cell turgor to prevent the damage from low water potential in ABA-independent signaling [7,11].

Three components play important roles in ABA signaling: pyrabactin resistance (PYR)/pyrabactin resistance-like (PYL)/regulatory component of ABA receptors (RCAR), protein phosphatase 2C (PP2C) and sucrose non-fermenting (SNF1)-related protein kinase 2 (SnRK2). In the absence of ABA, PP2Cs physically interact with SnRKs, which hinders SnRKs to phosphorylate down-stream transcription factors to activate ABA-responsive gene expression. In the presence of ABA, ABA receptors PYR1/PYL/PCAR bind with ABA and inhibit the activity of PP2Cs. This leads to the release of SnRKs and causes phosphorylation of ABF/ABRE/ABI5 bZIP family transcription factors which further activate ABA-induced gene expression [12,13,14].

There are nine members of clade A protein phosphatase 2C in Arabidopsis. Among them, six PP2Cs are involved in ABA signaling: ABI1, ABI2, ABA Hypersensitive Germination1 (AHG1), AHG3/AtPP2CA, Hypersensitive to ABA1 (HAB1), and HAB2 [15,16,17,18,19,20,21,22,23,24,25,26]. The other three PP2Cs, i.e., Highly ABA-Induced1 (HAI1), HAI2 and HAI3, have relatively less impact on ABA sensitivity phenotypes, but play an important role in dehydration resistance. The HAI PP2C mutants showed enhanced proline and osmoregulatory solute accumulation at low water potential, while other clade A PP2Cs had less effect on these drought resistance related traits. HAI1 selectively interacts with monomer-type PYLs including PYL5, PYL7, PYL8, PYL9, and PYL10, while does not interact with dimeric ABA receptors PYR1, PYL1, and PYL2. Several studies provided evidence that overexpression of PYL5, PYL8 or PYL9 obviously enhances plant tolerance to drought stress [3,27,28]. PP2Cs probably have undergone a functional diversification, for instance, HAI1, HAI2, and HAI3 mainly regulate dehydration resistance, while other members of clade A PP2Cs participate in ABA signaling [7].

ABA receptors are regulated by ubiquitination-mediated degradation via the 26S proteasome [29,30,31]. Thus far, three E3 ubiquitin ligases have been reported to be involved in this process. *Arabidopsis thaliana* DET1, DDB1-ASSOCIATED1 (DDA1), one component of the COP10-DET1-DDB1 (CDD)-related complex, provided substrate specificity for the CULLIN4-RING E3 ubiquitin ligases (CRL4s) by interacting with ABA receptors PYL4, PYL8 and PYL9. DDA1 promoted the degradation of PYL8. All the components in CDD complex negatively regulate ABA-mediated developmental responses [30]. A single-subunit RING-type E3 ubiquitin ligase RING FINGER OF SEED LONGEVITY1 (RSL1) interacted with the PYL4 and PYR1 ABA receptors at plasma membrane. RSL1 promoted PYL4 and PYR1 degradation in vivo. Overexpression of RSL1 caused plants to be insensitive to ABA while RNAi expression of RSL1 conferred increased ABA sensitivity [29]. RCAR3-INTERACTING F-BOX PROTEIN1 (RIFP1) interacted with PYL8/RCAR3. Overexpression of RIFP1 reduced ABA sensitivity while the *rifp1* mutants showed enhanced sensitivity to ABA [31].

According to the mechanism of action and the presence of specific domains, the E3 ubiquitin ligases were divided into four categories, RING, HECT, F-box, and U-box [32,33]. Sixty-four U-box genes were predicted in the *Arabidopsis* genome [34]. These U-box proteins play vital role in diverse developmental processes, especially in response to abiotic stress [35,36,37,38,39,40,41]. PUB22 and PUB23 are homologous plant U-box E3 ubiquitin ligases that share 77% identity in amino acid sequence. The transcript level of PUB22 and PUB23 were induced by abiotic stresses but not by ABA. Transgenic plants overexpressing PUB22 or PUB23 showed hypersensitivity to drought stress, while the *pub22 pub23* double mutants displayed enhanced tolerance to drought. RPN12a, a subunit of the 19S regulatory particle (RP) in the 26S proteasome, interacted with PUB22 and PUB23 in vitro and in vivo. RPN12a was highly ubiquitinated in transgenic plants overexpressing PUB22, suggesting that PUB22 and PUB23 negatively regulate drought tolerance probably by ubiquitination of RPN12a in *Arabidopsis* [35]. Additionally, RPN6, another subunit of the 19S RP, interacts with PUB22 and PUB23. PUB22 and PUB23 were able to ubiquitinate RPN6 in vitro and mediate the stability of RPN6 [42]. Expression of PUB19 was induced by abiotic stresses, such as drought, salt, cold and ABA. Overexpression of PUB19 resulted in insensitivity to ABA during seed germination and reduced ABA-induced stomatal closing. RNAi expression of PUB19 conferred the reverse phenotype, implying that PUB19 negatively regulates ABA signaling [37]. A negative regulator in ABA signaling, ABI1, interacted with PUB12 and PUB13. Ubiquitination of ABI1 by PUB12 and PUB13 occurred only when ABI1 interacted with ABA receptors in vitro. Additionally, *abi1-3* loss-of-function mutation could rescue the ABA-insensitive phenotype of *pub12 pub13*. PUB12 and PUB13 regulate ABA signaling by mediating ABI1 protein stability [36].

Here, we found that the transgenic plants overexpressing PYL9 showed enhanced drought tolerance. The PYL9 protein was degraded by the 26S proteasome while osmotic stress could stabilize PYL9. In yeast two-hybrid assay, we found PYL9 interacted with the plant U-box E3 ubiquitin ligase PUB22 and its highly homologous protein PUB23. We further confirmed that PYL9 interacted with PUB22 and PUB23 in vitro and in vivo. The *pub22 pub23* double mutants showed decreased PYL9 degradation, while overexpression of PUB22 led to the increased degradation of PYL9. Our biochemical assay is consistent with the previous finding that the *pub22 pub23* double mutant showed improved tolerance to drought [35]. Additionally, PUB22 and PUB23 interact with other ABA receptors such as PYL5, PYL7 and PYL8. These results suggest that PUB22 and PUB23 negatively regulate drought tolerance probably in part by modulating the stability of ABA receptors.

## 2. Results

### 2.1. Overexpression of PYL9 Confers Drought Tolerance

**Figure 1:**
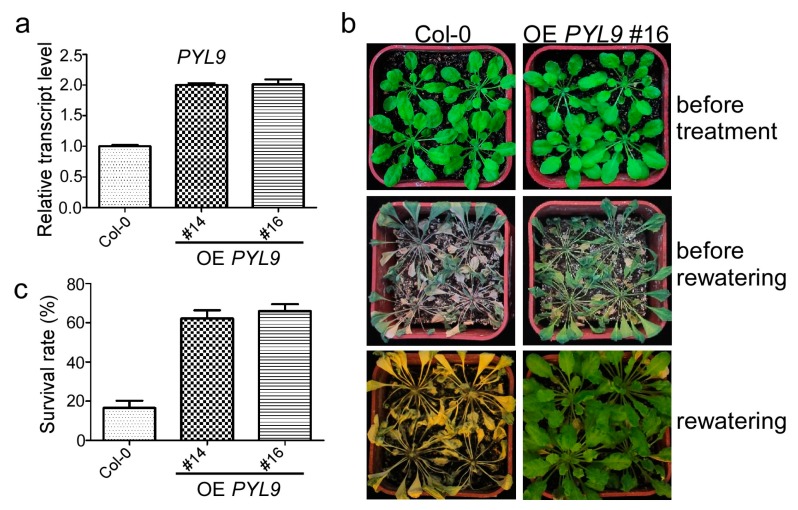
Overexpression of *PYL9* enhanced drought tolerance in plants. (**a**) Relative transcript level of *PYL9* in two transgenic lines (#14 and #16) harboring *Myc-PYL9*. Data are the means ± standard errors (*n* = 3). (**b**) Overexpression of *PYL9* confers drought tolerance in *Arabidopsis*. Four-week-old transgenic plants (upper) were treated by withholding water for 30 days (middle) and then rewatered. The representative images were taken after the plants were re-watered for 10 days (lower). (**c**) The percentage of surviving plants after re-watering for ten days. Data are the means ± standard errors (*n* = 24).

The *Arabidopsis* genome contains 14 genes encoding the PYR1/PYL/PCAR family ABA receptors, which play crucial roles in response to drought and other abiotic stresses [3]. In order to elucidate the function of PYL9, a less characterized ABA receptor, in drought stress, we generated transgenic plants harboring Myc-PYL9 driven by the CaMV35S promoter. The quantitative real-time PCR results showed that the transcript level of PYL9 was approximately two-fold higher in two representative transgenic lines overexpressing PYL9 (#14 and #16) compared with the wild-type (Figure 1a). In order to determine whether sustained transcriptional upregulation of PYL9 affects drought tolerance, we exposed the wild-type and the transgenic plants to dehydration condition by withholding water. There was no significant difference among them when grown under well-watered conditions (Figure 1b). After drought treatment for 30 days, both the wild-type and the transgenic lines displayed severe wilting. However, after rewatering for 10 days, we found that the transgenic plants expressing Myc-PYL9 showed a significantly higher survival rate than wild-type (Figure 1b,c). The survival rate of the transgenic line #14 and #16 was approximately 62.2% and 65.9%, respectively. However, the wild-type plants showed a much lower survival rate of approximately 16.6% (Figure 1c). Similar results were observed in a previous study [3]. We selected one transgenic line to show its drought tolerant phenotype (Figure 1b). These results showed that overexpression of PYL9 enhanced the tolerance of plants to drought stress.

### 2.2. PYL9 Is Degraded by the 26S Proteasome and Accumulates under Osmotic Stress

**Figure 2:**
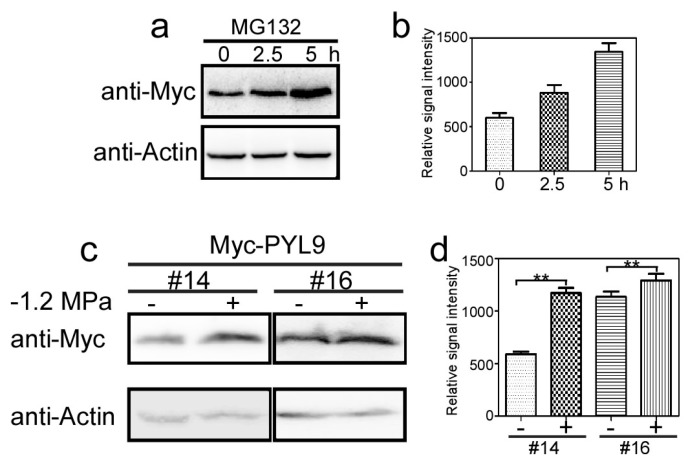
PYL9 was degraded by the 26S proteasome and osmotic stress increased PYL9 accumulation. (**a**) The proteasome inhibitor MG132 stabilized Myc-PYL9. Ten-day-old transgenic seedlings were treated with or without (mock) 50 μM MG132 for different time period. The bands detected by anti-Actin were used as loading control [44,45]. (**b**) Quantitative evaluation of the Myc-PYL9 protein level in (**a**). Data are the means ± standard errors (*n* = 3). (**c**) Osmotic stress increased the accumulation of Myc-PYL9. Seedlings of two T_3_ transgenic lines (#14 and #16) were grown on −1.2 MPa PEG-infused plates for three hours. The bands detected by anti-Actin were used as loading control. (**d**) Quantitative evaluation of the Myc-PYL9 protein level in (**c**). Independent experiments were repeated three times with the similar results. Data are the means ± standard errors (*n* = 3). Asterisks indicate significant differences (*p* < 0.05).

Several ABA receptors interact with different E3 ubiquitin ligases and are degraded by the 26S proteasome [29,30,31]. Although PYL9 interacted with DDA1 in yeast and may be substrate of the CRL4 ubiquitin ligase [30], it is still unclear whether PYL9 protein is degraded by the 26S proteasome. Here, we treated the transgenic plants overexpressing Myc-PYL9 with the proteasome inhibitor MG132 for different time (Figure 2a). Immunoblotting with anti-Myc antibody showed that Myc-PYL9 protein was accumulated in the MG132-treated samples at indicated time points compared with the mock treatment (Figure 2a,b). When the samples were treated with MG132 for 2.5 h or 5 h, the protein level of Myc-PYL9 was 1.6- or 2.4-fold higher than that in the sample without MG132 treatment (Figure 2b). Since overexpression of PYL9 enhanced drought tolerance in plants, we wanted to know whether PYL9 protein level was regulated by drought or osmotic stress. The transgenic plants harboring Myc-PYL9 were grown on 1/2 MS medium for ten days, and then transferred to −1.2 MPa PEG-infused plates for three hours [43] to impose low water potential stress that mimics osmotic stress. Osmotic stress obviously enhanced the Myc-PYL9 accumulation compared with the untreated samples in two transgenic lines (#14 and # 16) (Figure 2c). When the transgenic plants were treated by low water potential stress, the protein level of Myc-PYL9 was accumulated more than two-fold higher in one transgenic line (#14) and slightly but significantly higher in the other transgenic line (#16) than the seedlings before treatment (Figure 2d). These results suggest that there may be certain E3 ubiquitin ligases that regulate PYL9 protein level in response to osmotic stress.

### 2.3. PYL9 Interacts with PUB22 and PUB23

**Figure 3:**
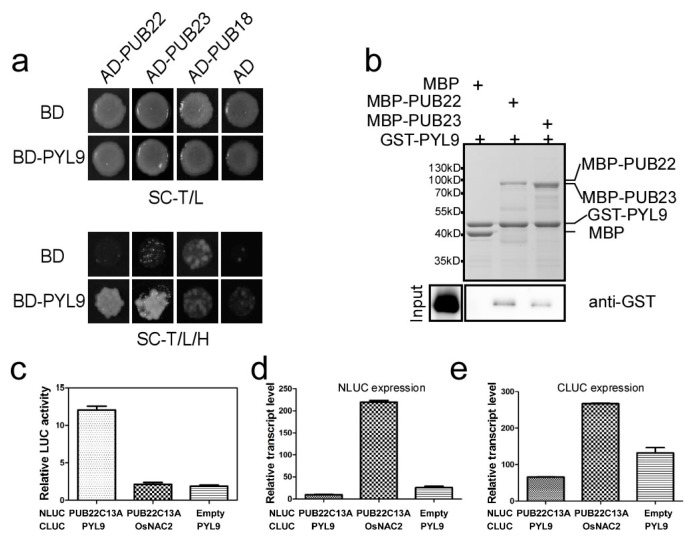
ABA receptor PYL9 interacts with two highly homologous plant U-box E3 ligases, PUB22 and PUB23. (**a**) PYL9 interacts with PUB22 and PUB23 in yeast two-hybrid assays. The growth of yeast transformed with the indicated constructs on selective plate is shown. Empty vectors (BD or AD) were used as negative controls. (**b**) PYL9 interacts with PUB22 and PUB23 in pull-down assays. Five micrograms MBP-PUB22, MBP-PUB23, or MBP bound with amylose resin were incubated with 1 μg GST-PYL9 for one hour at room temperature. Five percent of the input proteins were run on the PAGE gel and visualized via coomassie brilliant blue staining (upper panel). After washing five times, the protein complex pulled down by resin was detected with anti-GST antibody (lower panel). (**c**) PYL9 interacts with PUB22C13A by firefly luciferase complementation imaging assay. The indicated combinations were co-transfected with the CaMV35S:GUS construct into tobacco leaves and incubated for five days. LUC activity was normalized with the GUS activity. Data are the means ± standard errors (*n* = 3). (**d**) The transcript level of *NLUC* in the combinations in (**c**). Data are the means ± standard errors (*n* = 3). (**e**) The transcript level of CLUC in the combinations in (**c**). Data are the means ± standard errors (*n* = 3).

To determine which E3 ubiquitin ligase targets PYL9 in response to osmotic stress, we performed yeast two-hybrid assays. Plant U-box E3 ubiquitin ligases play diverse roles in *Arabidopsis* and several members such as PUB18, PUB22 and PUB23 have been shown to be involved in ABA-dependent or ABA-independent signaling in response to drought stress [38]. Thus, we first examined whether PYL9 interacts with these PUBs in yeast two-hybrid assay. The combination of AD-PUB18, AD-PUB22, AD-PUB23, and empty AD vector with BD-PYL9 or empty BD vector were co-transformed to the yeast cells. All of the clones grew well on the SC medium lacking Tryptophan and Leucine (SC-T/L). However, only the combination of PUB22 with PYL9 and PUB23 with PYL9 grew well on the SC medium lacking Tryptophan, Leucine and Histidine (SC-T/L/H), while the combination of PUB18 with PYL9 could not grew well (Figure 3a), indicating that PYL9 interacted with PUB22 and PUB23 in yeast.

To further confirm the interaction between PUB22 or PUB23 and PYL9, in vitro pull-down assay was performed. The *PUB22* and *PUB23* coding sequence were cloned into the *pCold-MBP* vector containing a maltose binding protein (MBP) tag, respectively. MBP-PUB22 or MBP-PUB23 fusion proteins were purified from the *Escherichia coli* strain BL21 (DE3) with amylose resin (BioLabs). The *PYL9* coding sequence was inserted into the *pCold-GST* vector with a glutathione S-transferase (GST) tag. GST-PYL9 was purified with Glutathione Sepharose^TM^4B resin (GE Healthcare). In vitro pull-down assays showed that GST-PYL9 was pulled down by MBP-PUB22 or MBP-PUB23 but not by the sole MBP tag (Figure 3b). These results further confirmed that PUB22 and PUB23 interact with PYL9.

To elucidate whether PUB22 interacts with PYL9 in vivo, we performed firefly luciferase complementation imaging assay [46]. According to previous studies, the C13A substitution in PUB22 amino acid sequence (PUB22C13A) produced an inactive mutant variant, which is unable to bind the ubiquitin conjugating enzyme E2 [39]. To avoid the potential degradation of PYL9 mediated by PUB22 which may prevent detection of the interaction between these two proteins, the *PUB22C13A* mutant cDNA was translationally fused to the N terminus of the NLUC fragment. The *PYL9* coding sequence was translationally fused to the C terminus of CLUC fragment. The *OsNAC2* gene (LOC_Os04g38720), encoding a NAC transcription factor in rice [47], was used as a negative control. *Agrobacterium* strains carrying *NLUC* and *CLUC* constructs were mixed and infiltrated into leaves of *N. benthamiana*. Five days later, leaves co-expressing different constructs were cut to detect the chemiluminescence signal with charged coupled device camera when the substrate of luciferase, luciferin, was sprayed on the tobacco leaves. However, no signal was detected. We proposed that the interaction between PUB22 and PYL9 may be weak. Thus, we applied a more sensitive method to detect the LUC activity with the Luciferase Assay System (Promega, Madison, WI, USA). The tobacco leaves transfected with different combinations were grinded with liquid nitrogen to isolate total protein. Equal quantities of plant extracts were used to detect the LUC activity. At the same time, the transcript level of *NLUC* and *CLUC* in each combination was measured to detect the transfection efficiency. The results showed that the LUC activity of leaves co-expressing *PUB22C13A* and *PYL9* was significantly higher than those co-expressing *PUB22C13A* and *OsNAC2* or empty *NLUC* and *PYL9* (Figure 3c), although the transcript level of *NLUC* and *CLUC* were lower in the combination of *PUB22C13A* and *PYL9* than that in the other two combinations (Figure 3d,e). These results suggested that PUB22 interacted with PYL9 in vivo.

### 2.4. PUB22 and PUB23 Co-Regulate the Stability of PYL9

**Figure 4:**
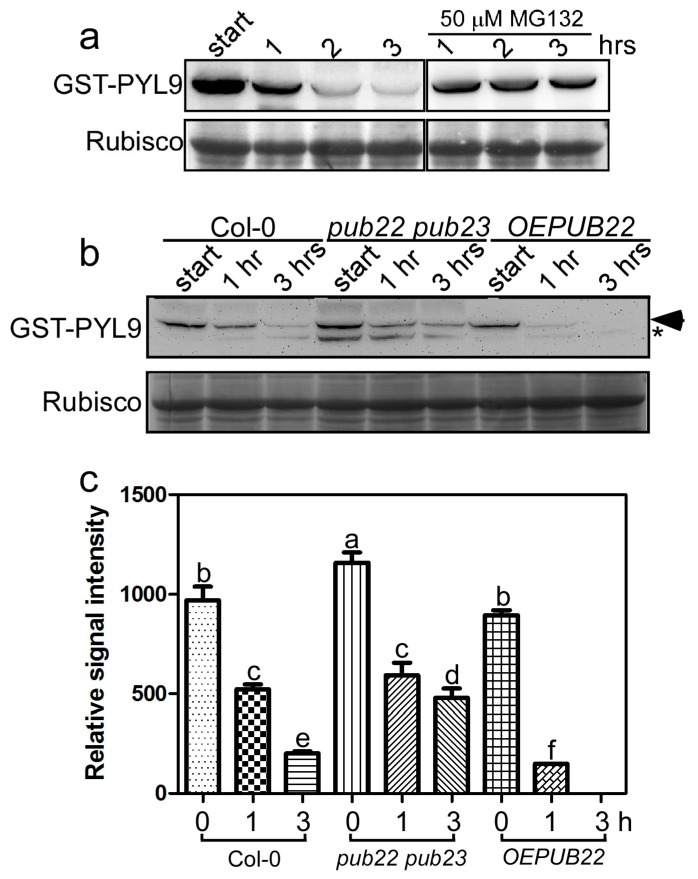
PUB22 and PUB23 regulate GST-PYL9 stability in cell-free degradation assay. (**a**) The degradation of GST-PYL9 in the extract from the wild-type at indicated time. MG132, a specific 26S proteasome inhibitor, was used as a proteasomal activity control. Coomassie brilliant blue (CBB) staining of Rubisco was used as loading control. (**b**) The degradation of GST-PYL9 in the wild-type, the *pub22 pub23* double mutant and the transgenic plants overexpressing *PUB22* (*OEPUB22*). The total protein isolated from ten-day-old seedlings were incubated with GST-PYL9 for the indicated hours. GST-PYL9 was detected with anti-GST antibody. CBB staining of Rubisco was used as loading control. Asterisk may represent truncated GST-PYL9 or unspecific band. (**c**) Quantitative evaluation of the GST-PYL9 protein level in (**b**). Independent experiments were repeated three times with the similar results. Data are the means ± standard errors (*n* = 3). Statistical significance was determined by a Student’s *t*-test; significant differences (*p* ≤ 0.05) are indicated by different lowercase letters.

PUB22 and its highly homologous protein PUB23 interact with PYL9 in vitro and in vivo. To understand the biological significance of this interaction, we conducted a cell-free degradation assay [48] to determine whether PUB22 and PUB23 affect the stability of PYL9 protein. The *pub22* (SALK_072621C) and *pub23* (SALK_063470C) mutant lines reported previously [35] were ordered from the Arabidopsis Biological Resource Centre (ABRC). We obtained the *pub22 pub23* double mutant by crossing the *pub22* and *pub23* single mutants. The mRNA transcripts of *PUB22* and *PUB23* were dramatically decreased in the *pub22 pub23* double mutants (Supplementary Materials, Figure S1). We also generated transgenic plants overexpressing *FLAG-PUB22* in which FLAG-PUB22 protein can be detected using anti-FLAG antibody (Supplementary Materials, Figure S2). The GST-PYL9 purified from *E. coli* was first incubated with total protein extract from the wild-type with at 22 °C for 1, 2 and 3 h. The mixture was then analyzed using immunoblotting with anti-GST antibody. With increase in the incubation time, the GST-PYL9 was gradually degraded, whereas the 26S proteasome inhibitor, MG132, obviously inhibited the degradation of GST-PYL9 (Figure 4a). This result demonstrated that degradation of PYL9 is due to proteasomal activity. Then GST-PYL9 was incubated with the total protein extracts from the *pub22 pub23* double mutant and the transgenic seedlings overexpressing *FLAG-PUB22* at 22 °C for 1 h or 3 h. The degradation of GST-PYL9 was more intense in the extract from transgenic seedlings overexpressing *PUB22* than in that from the wild-type after incubating for both 1 h and 3 h. On the other hand, the degradation of GST-PYL9 became slower in the extract from the *pub22 pub23* double mutant than in that from the wild-type, especially after incubating for 3 h (Figure 4b,c). These results suggest that PUB22 and PUB23 would likely modulate the degradation of PYL9.

**Figure 5:**
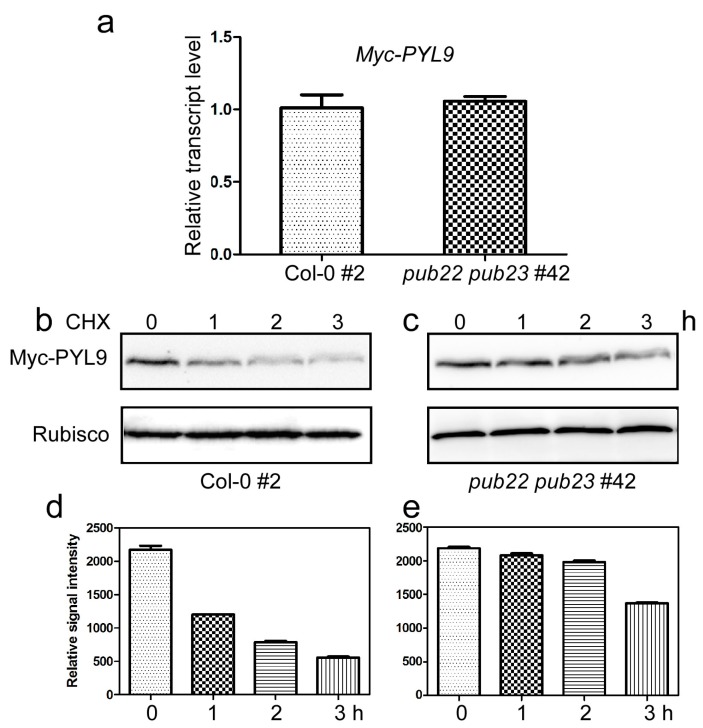
Myc-PYL9 is stabilized in the *pub22 pub23* double mutant. (**a**) The transcript level of *Myc-PYL9* in the transgenic plants in Col-0 (#2) or *pub22 pub23* (#42) background. Data are the means ± standard errors (*n* = 4). (**b**,**c**) Degradation rate of *Myc-PYL9* in Col-0 (**b**); and the *pub22 pub23* double mutant (**c**). Ten-day-old transgenic seedlings were incubated in liquid MS medium containing 50 μM MG132 for 16 h and washed five times before being transferred to liquid medium with 100 μM cycloheximide (CHX). Proteins were isolated at the indicated time points and detected with anti-Myc antibody. Anti-Actin was used as loading control. (**d**) Quantitative evaluation of the Myc-PYL9 protein level in (**b**). (**e**) Quantitative evaluation of the Myc-PYL9 protein level in (**c**). Independent experiments were repeated three times with the similar results. Data are the means ± standard errors (*n* = 3).

To further examine the effect of PUB22 and PUB23 on the stability of PYL9 in vivo, we carried out the following experiments. The *Myc-PYL9* fusion gene driven by the *CaMV35S* promoter was transformed into the wild-type Col-0 and the *pub22 pub23* double mutant, respectively. Two transgenic lines showing comparable transcript level of *Myc-PYL9* were identified from each background, i.e., line #2 in the wild-type background (Col-0 #2) and line #42 in the *pub22 pub23* double mutant background (*pub22 pub23* #42, Figure 5a). We then compared the stability of Myc-PYL9 protein in these two lines with different background. After treatment with cycloheximide, a protein synthesis inhibitor that prevents de novo protein synthesis, seedlings were collected at different time points for immunoblot analysis using anti-Myc antibody. The Myc-PYL9 protein was degraded rapidly with a half-life of about 1 h in the wild-type (Figure 5b,d). As expected, the degradation of Myc-PYL9 in the *pub22 pub23* double mutant was significantly prolonged with a half-life of approximately 3 h (Figure 5c,e). These results provided strong evidence that PYL9 is destabilized by PUB22 and PUB23 in vivo.

### 2.5. Expression Pattern of PUB22, PUB23 and PYL9

**Figure 6:**
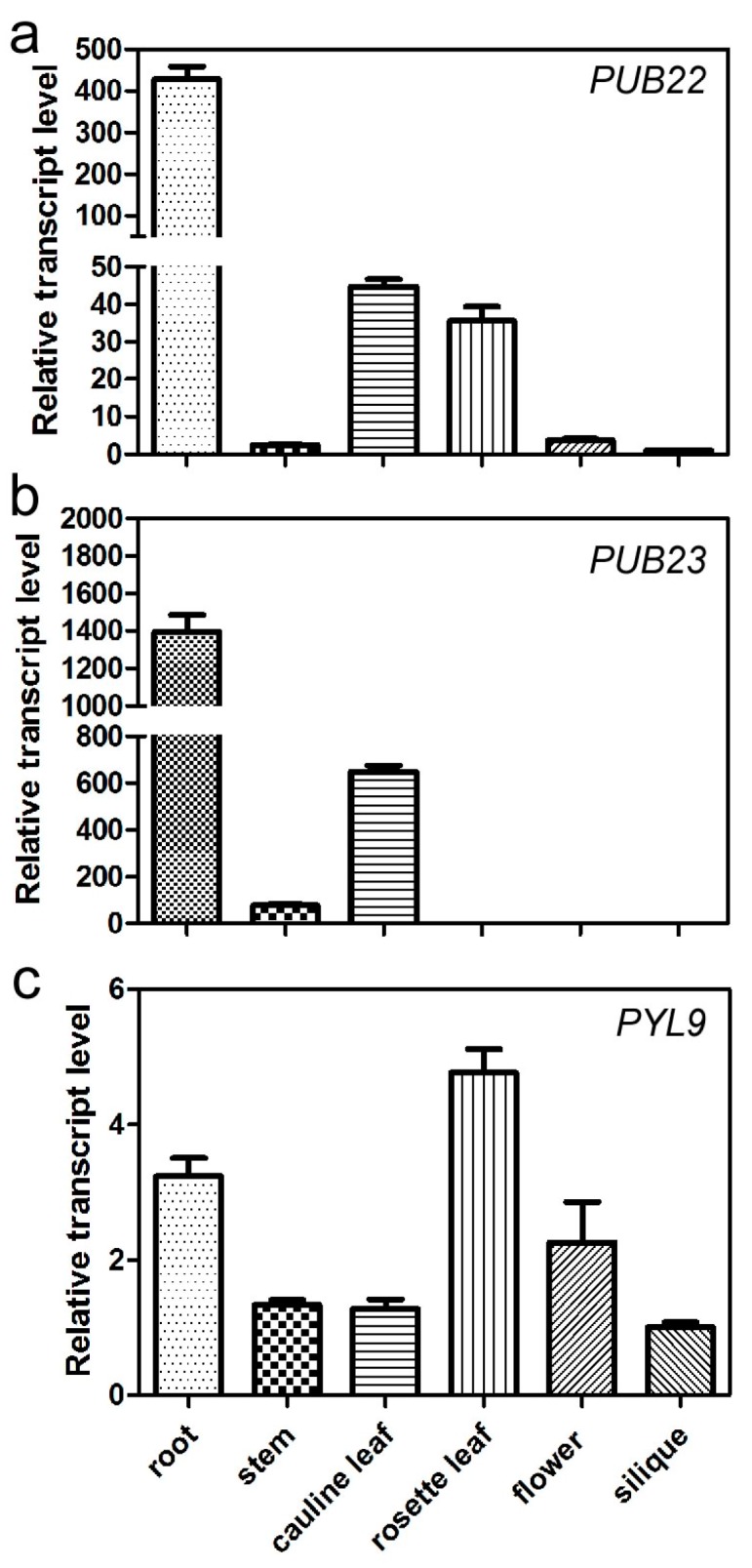
Expression patterns of *PUB22*, *PUB23* and *PYL9*. (**a**–**c**) Quantitative real-time PCR was used to determine the expression of *PUB22* (**a**); *PUB23* (**b**); and *PYL9* (**c**), in root, stem, cauline leaf, rosette leaf, flower, and silique of the *Arabidopsis* wild-type Col-0. Data are the means ± standard errors (*n* = 4).

To reveal the expression pattern of *PUB22, PUB23* and *PYL9*, we performed the quantitative real-time PCR using mRNA from different organs of the *Arabidopsis* wild-type Col-0, including root, stem, cauline leaf, rosette leaf, flower, and silique. The results showed that *PUB22* and *PUB23* were mainly expressed in the root. The transcript level of *PUB22* in root is approximately 100-fold higher than that in stem, flower or silique, and about 10-fold higher than that in cauline or rosette leaves (Figure 6a). At the same time, we found that *PUB23* also has strong expression in roots compared with other organs. The transcript level of *PUB23* in roots is almost 1000-fold higher than that in rosette leaf, flower or silique, about seven-fold higher than that in stem and approximately two-fold higher than that in cauline leaf (Figure 6b). *PYL9* has higher expression in root, rosette leaf, and flower compared with stem, cauline leaf, and silique (Figure 6c). The quantitative real-time PCR analysis revealed that PUB22 and PUB23 have strong expression in the root and *PYL9* also has expression in the root tissue.

### 2.6. PUB22 Interacts with PYL5, PYL7, PYL8 and PYL9

**Figure 7:**
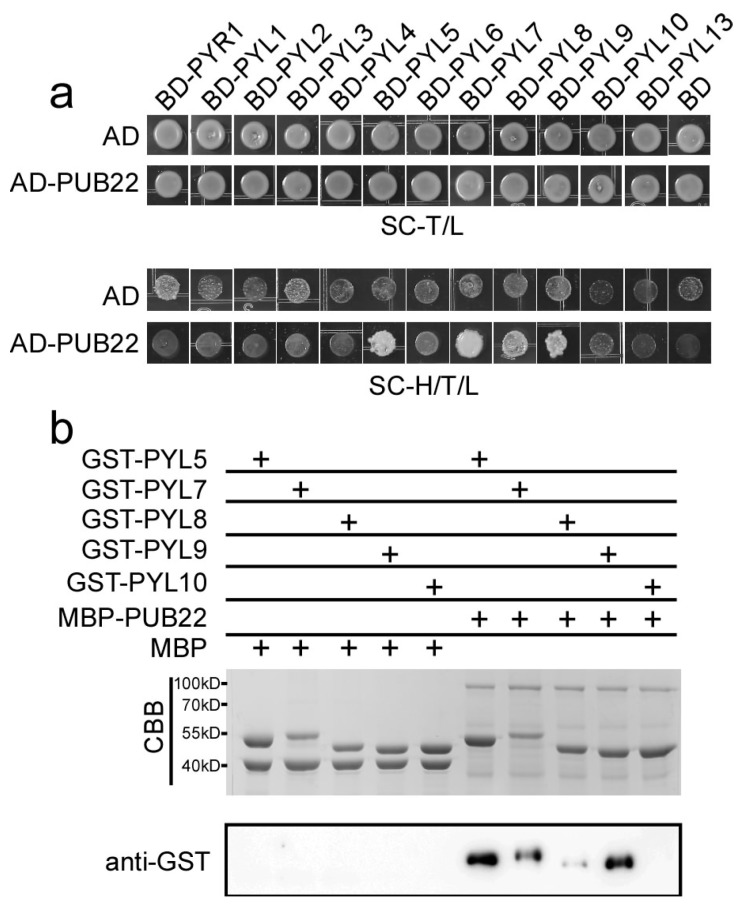
PUB22 interacts with PYL5, PYL7, PYL8, and PYL9. (**a**) PUB22 interacts with PYL5, PYL7, PYL8, and PYL9 in yeast two-hybrid assay. The combinations of PUB22 with different PYLs were grown on the SC medium lacking Tryptophan and Leucine (SC-T/L) or Tryptophan, Leucine and Histidine (SC-H/T/L). (**b**) PUB22 interacts with PYL5, PYL7, PYL8, and PYL9 in the pull-down assay. MBP-PUB22 and MBP bound with Amylose resin were incubated with GST-PYL5, GST-PYL7, GST-PYL8, or GST-PYL9, separately. Five percent of the input proteins were run on the PAGE gel and visualized via coomassie brilliant blue (CBB) staining (upper panel). The protein complex pulled down by resin was detected with anti-GST antibody (lower panel).

In order to examine whether PUB22 interacts with other ABA receptors, we performed yeast two-hybrid assay. We cloned all the ABA receptors except PYL11 and PYL12 into the *pGBKT7* vector, in which the receptor coding sequences were translationally fused with the GAL4 DNA binding domain. The resulting constructs were co-transformed with *AD-PUB22* or the empty *AD* vector. All the yeast clones grew well on the SC medium lacking Tryptophan and Leucine. However, only the co-transformants of PYL5, PYL7, PYL8 and PYL9 with PUB22 grew well on the selective SC medium lacking Tryptophan, Leucine and Histidine (Figure 7a).

To further confirm the interaction between PYL5, PYL7, PYL8 or PYL9 and PUB22, in vitro pull-down assay were performed. The PYL5, PYL7, PYL8, PYL9 and PYL10 coding sequence were translationally fused with GST tags and fusion proteins were purified from DE3 cells using Glutathione Sepharose^TM^4B resin. Eluted proteins were incubated with the MBP-PUB22 resin or the MBP resin at room temperature for 1 hour. Immunoblotting with anti-GST antibody showed that GST-PYL5, GST-PYL7, GST-PYL8 and GST-PYL9 could be pulled down by MBP-PUB22. Consistent with the yeast two-hybrid assay, the negative control GST-PYL10 was not pulled down by MBP-PUB22. These results suggest that PUB22 interacts with PYL5, PYL7, PYL8 and PYL9 in vitro (Figure 7b).

## 3. Discussion

Although great progress on the ABA signaling pathways and its function in response to drought has been made, the role of various ABA receptors in drought tolerance still needs to be elucidated. In this study, we have revealed the function of one of the ABA receptor *PYL9* in regulating drought tolerance in *Arabidopsis*. The PYL9 protein level was regulated by the 26S proteasome and osmotic stress increased the accumulation of PYL9. Plant U-box E3 ubiquitin ligases PUB22 and PUB23 interact with PYL9 and regulate the stability of the PYL9 protein. Overexpression of *PUB22* enhanced the degradation of PYL9, whereas PYL9 was degraded much more slowly in the *pub22 pub23* double mutant. In previous studies, PUB22 and PUB23 were reported to be negative regulators in drought responses. The *pub22 pub23* double mutant was more tolerant to drought although its ABA-mediated stomatal movements remained similar to those of the wild-type plants [35,38]. By contrast, *PUB12*, *PUB13* and *PUB19* are mainly involved in ABA response [36,37]. Among nine clade A protein phosphatases, the HAI PP2C mutants accumulated more proline and osmoregulatory solute but had less effect on ABA sensitivity, in contrast to other clade A PP2Cs. This suggests that HAI1, HAI2, and HAI3 are mainly involved in dehydration resistance but not ABA signal transduction. HAI1 interacts with the monomer-type PYLs including PYL5, PYL7 and PYL10 [7]. In the present study, we found that these PYLs interact with PUB22 except PYL10 (Figure 7). Moreover, SnRK2s, another core component in ABA signaling, probably functions in the convergence between ABA-dependent and -independent pathway in response to osmotic stress [49]. All these results imply that there are putative PUBs-PYLs-PP2Cs-SnRKs signaling cascade in ABA-independent pathway in response to osmotic stress. Because osmotic stress always accompanies with ABA biosynthesis, it is difficult to distinguish ABA-dependent and ABA-independent pathway in the PUBs-PYLs-PP2Cs-SnRKs signaling cascades.

In our study, we detect the interaction between PUB22 and PYL9 with firefly luciferase complementation imaging assay. We also performed a bimolecular fluorescence complementation (BiFC) and coimmunoprecipitaton analysis to confirm whether PUB22 interact with PYL9 in vivo. The mutated coding sequence of *PUB22* (*PUB22C13A*) and *PYL9* were cloned into *pSPYCE(M)* and *pSPYNE173* plasmid [50], respectively. The combination of *pSPYCE(M)-PUB22C13A* with *pSPYNE173-PYL9* and *pSPYCE(M)-PUB22C13A* with *pSPYNE173* empty vector were transiently expressed in the tobacco leaves. After 5 days incubation, we could detect the strong YFP signal in the tobacco leaves that the combination of *pSPYCE(M)-PUB22C13A* with *pSPYNE173* empty vector was transfected. Thus, we proposed that PUB22 oligomerization [51] may result in the false positive results. Additionally, coimmunoprecipitation analysis was carried out. *FLAG-PUB22* and *Myc-PYL9* were co-transformed into the *Arabidopsis* mesophyll protoplasts. Although we could detect the expression of FLAG-PUB22 and Myc-PYL9 as input, we could not detect the interaction between them. Such a limitation might be because PUB22 and PUB23 are mainly expressed in the root, whereas these experiments were performed in leaves. It is likely that additional factors expressed in roots might be required for such interaction. We also crossed the transgenic plants harboring *FLAG-PUB22* with the transgenic plants expressing *Myc-PYL9* and used the F1 progeny seedlings in the coimmunoprecipitation assay. We could not get the positive results, either. According to these results, we proposed that the instability of PUB22 [51] maybe affect the interaction with PYL9 or the interaction between PUB22 and PYL9 was too weak to be detected with immunoblotting. When plants suffer from drought stress, it is the roots that first sense the low water potential. Quantitative real-time PCR showed that *PUB22* and PUB23 were mainly expressed in the roots (Figure 6a,b), which is consistent with its roles in drought response rather than ABA-mediated responses. *PYL9* was also expressed in the root (Figure 6c). The overlapping expression pattern of *PUB22*, *PUB23* and *PYL9* in the roots may facilitate the response to osmotic stress as quick as possible.

Thus far, three kinds of E3 ubiquitin ligases were reported to interact with ABA receptors and regulate their protein stability. DDA1, as part of the CDD complex, provides substrate specificity for the CRL4 E3 ubiquitin ligase, through interacting with PYL8 in the nucleus [30]. The F-box protein RIFP interacts with PYL8 also in the nucleus [31]. Interaction of the RING-type E3 ubiquitin ligase RSL1 with PYR1 and PYL4 is localized to plasma membrane [29]. In this study, we reported that another kind of E3 ubiquitin ligases, i.e., plant U-box E3 ubiquitin ligases PUB22 and PUB23, interact with ABA receptors PYL5, PYL7, PYL8 and PYL9. PUB22 and PUB23 were previously shown to be mainly localized in the cytoplasm [35]. It is likely that the stability of ABA receptors were finely tuned by different E3 ubiquitin ligases in different subcellular compartments.

The transgenic plants overexpressing *Myc-PYL9* not only showed drought tolerant phenotype, but also displayed increased tolerance to osmotic stress (Figure S3). Seedlings of the wild-type and the *Myc-PYL9* transgenic plants were grown on MS medium for seven days and transferred on the MS medium with or without different concentrations of mannitol for 12 days. There was no distinguishable difference between the wild type and the transgenic plants in fresh weight when grown on the MS medium. With increase in the concentration of mannitol, fresh weight of the wild-type was significantly decreased than the transgenic plants. Especially, the fresh weight of the wild-type was clearly reduced on medium containing 250 mM mannitol (Figure S3). Thus, the transgenic plants with *Myc-PYL9* were more tolerant to mannitol than the wild-type. In addition, we also examined seed germination of the wild-type and transgenic plants harboring *Myc-PYL9*. The results showed that the seeds of two independent transgenic lines containing *Myc-PYL9* are more sensitive to ABA than the wild-type when grown on medium with 0.5 μM ABA (Figure S4). In our study, PUB22 and PUB23 interact with PYL9 and regulate the stability of PYL9. The *pub22 pub23* double mutant showed drought tolerant phenotype [38]. We speculate that PUB22 and PUB23 are involved in drought tolerance probably partially by regulating the stability of PYL9.

However, the seed germination and stomatal movement of the *pub22 pub23* double mutant are insensitive to ABA [38]. We suggest the following possibilities to explain this phenomenon. Firstly, the *pub22 pub23* double mutant might gain drought tolerance via the role of PYL9 in certain ABA-independent process. As we know, PYL9 takes part in many aspects of plant growth and development. Transgenic plants overexpressing *PYL9* not only showed drought tolerance, but also displayed increased tolerance to osmotic stress (Figure S3). Moreover, PYL9 and PYL8 play an important role in regulating lateral root growth via direct interaction with MYB transcription factors [52]. A putative PYLs-PP2Cs-SnRKs signaling cascade has also been proposed in the ABA-independent pathway in response to osmotic stress [53] Secondly, although the degradation of PYL9 was reduced in the *pub22 pub23* mutant, the protein level of PYL9 may not be high enough for the double mutant to show the ABA sensitivity phenotype, as transgenic seeds overexpressing *PYL9* did. Originally, we had planned to compare the protein level of PYL9 in the wild-type, the *pub22 pub23* double mutant and transgenic plants overexpressing *PYL9* using anti-PYL9 antibody. However, the anti-PYL9 antibody we developed recognized not only the PYL9 protein, but also PYL7, PYL8 and PYL10 owing to their high homology. Thirdly, PYL9, PUB22 and PUB23 proteins may regulate many other targets that function in various processes. Some of these targets may prevent the *pub22 pub23* double mutant from showing the ABA sensitive phenotype. Further study will help us to uncover the intrinsic mechanism.

PYL9 protein was accumulated when treated by low water potential (Figure 2c). ABA treatment also enhanced PYL9 protein stability (Figure S5). Certainly, we could not exclude the possibility that osmotic stress increased PYL9 protein level by promoting ABA biosynthesis. PYL9 complexes not only with CRL4-DDA1, which is involved in ABA signaling [30], but also with PUB22 and PUB23 that are only involved in dehydration resistance [35,38]. These results suggest that PYL9 might be a crucial integration point between ABA-dependent and ABA-independent signaling in response to drought stress.

## 4. Materials and Methods

### 4.1. Plant Growth Condition and Stress Treatment

Four-week-old plants grown in the greenhouse under short day conditions were treated by withholding water for 30 days until most of the plants showed severe wilting. Then the plants were re-watered. Ten days later, the plants with green leaves were counted as survival plants and the plants with no green leaves were counted as dead plants. For osmotic stress treatment, −1.2 MPa PEG-infused plates were prepared [54,55]. Ten-day-old seedlings were transferred to −1.2 MPa PEG-infused plates for 3 h and harvested for protein isolation.

### 4.2. Generation of Overexpression Plants

To get overexpression plants, the cDNA of *PYL9* and *PUB22* were cloned into the *pCAMBIA1307-Myc* or *pCAMBIA1307-FLAG* [56] vectors between *Sal*I and *Kpn*I sites, respectively. The primers were listed in Supplementary Materials Table S1. The constructs were verified by sequencing and transformed into *Agrobacterium* strain GV3101. Then the *pCAMBIA1307-Myc-PYL9* construct was introduced into *Arabidopsis thaliana* ecotype Columbia (Col-0) and the *pub22 pub23* double mutants, and the *pCAMBIA1307-FLAG-PUB22* construct was transformed into Col-0 using the floral dip transformation method [57].

### 4.3. Quantitative Real-Time PCR

Total RNA was extracted from *Arabidopsis* seedlings and different tissues or tobacco leaves with Trizol reagent (Invitrogen, Carlsbad, CA, USA). Ten micrograms total RNA were treated with DNase I (TaKaRa Bio Inc., Kusatsu, Japan) to remove any contamination of genomic DNA. Subsequently, the resulting 1 μg total RNA was used in reverse transcription with M-MLV reverse transcriptase (TaKaRa). The quantitative real-time PCR was performed using SYBR premix Ex Taq (TaKaRa) on the ABI7500 instrument with gene-specific primers. All the primers were listed in supplementary materials Table S2.

### 4.4. Yeast Two-Hybrid Assay

To detect the interaction between ABA receptors (PYLs) and U-box E3 ubiquitin ligases (PUBs), the *PYLs* cDNA were amplified and cloned into the *pGBKT7* vector at the *EcoR*I and *BamH*I sites. The coding sequence of the *PUBs* genes were inserted into the *pGADT7* vectors between *EcoR*I and *BamH*I sites. The primers were listed in Table S1. The *BD-PYLs* constructs were introduced into the yeast strain AH109, and *AD-PUBs* were transformed into the yeast strain Y187 using the small-scale LiAc yeast transformation procedure (Coolaber Tech Inc., Beijing, China). Yeast two-hybrid screening was performed according to manufacturer’s instructions for the MATCHMARKER Two-Hybrid System (Clontech Laboratories, Mountain View, CA, USA) and diploid yeasts were screened on synthetic complete medium lacking tryptophan and leucine (Coolaber Tech Inc., Beijing, China).

### 4.5. In Vitro Pull-Down Assays

To determine the interaction between PYLs and PUBs, the *PYLs* cDNA were amplified by PCR and translationally fused to the C terminus of GST tag in the *pCold-GST* vectors [58] between *EcoR*I and *Sal*I sites. The fusion protein were purified with Glutathione Sepharose^TM^4B (GE Healthcare, Piscataway, NJ, USA) resin and eluted with reduced glutathione. The coding sequences of *PUBs* were cloned into the C terminus of MBP in the *pCold-MBP* vector [59] at *EcoR*I and *Sal*I sites. The primers were listed in Supplementary Materials Table S1. The protein was purified with amylase resin (New England Biolabs, Ipswich, MA, USA). For pull-down assay, 5 μg of GST-PYLs were incubated with 1 μg MBP-PUBs on amylase resin for 1 h at room temperature in 100 μL of binding buffer (20 mM Tris-HCl, pH 7.2, 10 mM MgCl_2_, and 2 mM DTT). After washing five times with binding buffer, the beads were resuspended in 100 μL loading buffer and 5 μL was analyzed by protein gel blotting using anti-GST antibody.

### 4.6. Firefly Luciferase Complementation Imaging Assay

To confirm the interaction between PYL9 and PUB22 in vivo, we performed firefly luciferase complementation imaging assay [46]. The coding sequence of *PUB22C13A* amplified by PCR using *pCold-MBP-PUB22C13A* as template was cloned into the *pCAMBIA1300-NLUC* vector at *Sal*I and *Xho*I sites, in which PUB22C13A was fused to the N terminus of NLUC. The cDNA of *PYL9* and negative control *OsNAC2* were inserted into the *pCAMBIA1300-CLUC* vector at *Kpn*I and *Sal*I sites, in which PYL9 and OsNAC2 was fused to the C terminus of CLUC. All the primers were listed in the Supplementary Materials Table S1. The combination of *PUB22C13A-NLUC* with *CLUC-PYL9* or *CLUC-OsNAC2* and the combination of empty *NLUC* with *CLUC-PYL9* were co-transfected into tobacco leaf and incubated for 5 days. Each combination was cotransfected with the CaMV35S:GUS construct, which was used as an internal control of the transformation efficiency. About 0.1 g cotransfected tobacco leaves were ground into powder with liquid N_2_ and dissolved in 100 μL 1× Cell Culture Lysis Reagent, followed by centrifugation for 15 min at 4 °C. Clear supernatant were used for LUC and GUS assay. The LUC activities were measured with the Luciferase Assay System (Promega) on the TriStar Multimode Microplate Reader LB 941 (Berthold Technologies, Oak Ridge, TN, USA) at once. The GUS activity was measured essentially as described previously [60]. LUC activity was normalized with GUS activity and the interaction level was expressed as the ratio of LUC to GUS.

### 4.7. Cell-Free Degradation Assay

Ten-day-old seedlings were harvested and ground to fine powder in liquid nitrogen. Total proteins were extracted in the degradation buffer containing 25 mM Tris-HCl, pH 7.5, 10 mM NaCl, 10 mM MgCl_2_, 4 mM phenylmethylsulfonyl fluoride (PMSF), 5 mM dithiothreitol (DTT), and 10 mM ATP as described [48]. Cell debris was removed by two 10 min centrifugation at 17,000× *g* at 4 °C. The supernatant was collected and protein concentration was determined using the Bradford protein assay. Five hundred micrograms of total protein extract were incubated with 100 ng of GST-PYL9 in 100 μL degradation buffer at 22 °C, and samples were collected at the indicated time point for determination of GST-PYL9 abundance by immunoblot assays.

### 4.8. MG132 and Cycloheximide Treatments

Ten-day-old transgenic *Arabidopsis* seedlings of *35S: Myc-PYL9* were transferred to liquid MS medium with or without 50 μM MG132 (Sigma-Aldrich Corporation, St. Louis, MI, USA) for 3 h. Treated seedlings were collected for immunoblot analysis using anti-Myc antibody. To compare the half-life of Myc-PYL9 in the wild-type and *pub22 pub23* double mutants, we followed the method described by Jung et al., 2015 [61]. The transgenic seedlings were pretreated with MG132 for 16 h and then washed five times before being transferred to liquid MS medium containing 200 mM cycloheximide (Sigma) to block de novo protein synthesis. The samples were harvested at the indicated time points and the total protein was isolated for immunoblotting using anti-Myc antibody.

## Supplementary Materials

Supplementary materials can be found at www.mdpi.com/1422-0067/18/9/1841/s1.

## Abbreviations

BiFC	Bimolecular Fluorescence Complementation
CBB	Coomassie Brilliant Blue
DE3	*Escherichia coli* strain BL21
GST	Glutathione *S*-transferase
MBP	Maltose binding protein
SC-T/L	SC medium lacking Tryptophan and Leucine
SC-T/L/H	SC medium lacking Tryptophan, Leucine and Histidine.
