# Supplementary material for: Arabidopsis E3 Ubiquitin Ligases PUB22 and PUB23 Negatively Regulate Drought Tolerance by Targeting ABA Receptor PYL9 for Degradation
Source: Int J Mol Sci. 2017 Aug 24;18(9):1841. doi: 10.3390/ijms18091841 (PMC5618490; doi:10.3390/ijms18091841)
Supplement: Supplementary file 1 [file ijms-18-01841-s001.pdf]

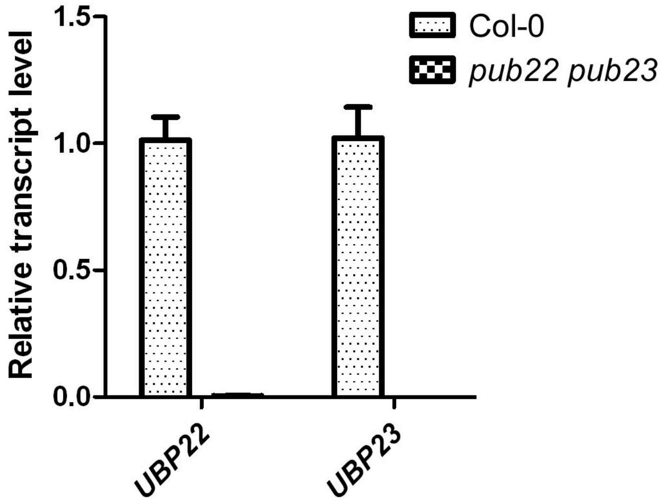

Supplementary Figure S1. Expression of *PUB22* and *PUB23* in the *pub22 pub23* double mutant. Quantitative real-time PCR determined the expression of *PUB22* and *PUB23* in *pub22 pub23* double mutant. The *pub22* (SALK_072621) and *pub23* (SALK_063470) single mutants[35]. Data are the means ± standard errors (*n* = 3).

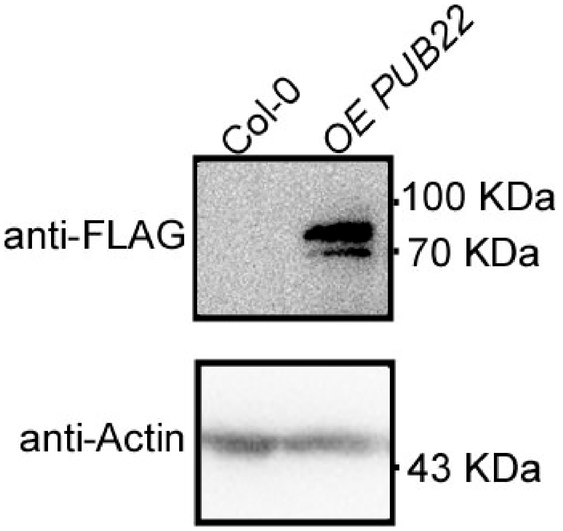

Supplementary Figure S2. The *expression* of *FLAG-PUB22* in transgenic plants. The expression of *FLAG-PUB22* in transgenic plants was analyzed by immunoblotting with anti-FLAG antibody.

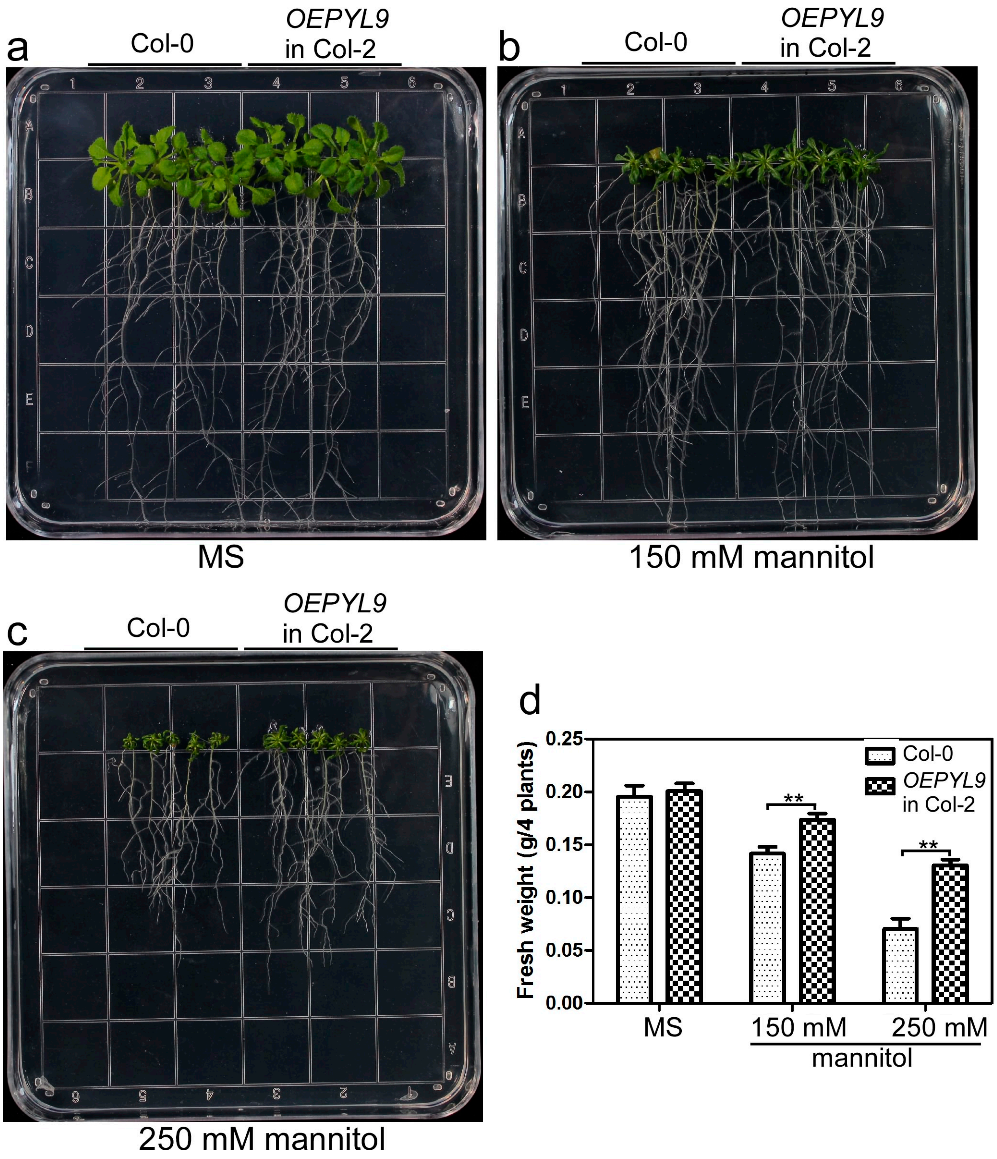

Supplementary Figure S3. The seedling growth of the wild-type and the transgenic seedlings harboring *Myc-PYL9* on MS with or without mannitol. (**a**), (**b**) and (**c**). Seven-day-old seedlings grown on MS medium were transferred to the medium with different concentration of mannitol for 12 days. (**d**) Quantitative evaluation of the fresh weight of seedlings in (**a**), (**b**) and (**c**). Asterisks indicate significant differences ($p < 0.05$).

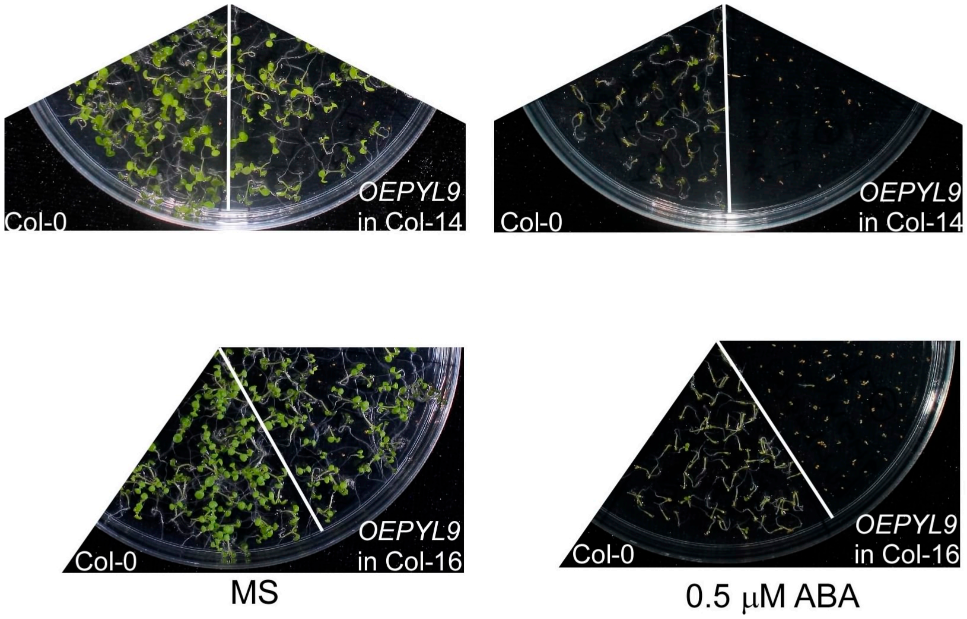

Supplementary Figure S4. The seeds germination of the wild-type and the independent transgenic seeds with *Myc-PYL9* on MS with or without 0.5 μM ABA. The seeds were vernalized at 4 °C for 3 days and grown on the chamber at 22 °C for 9 days.

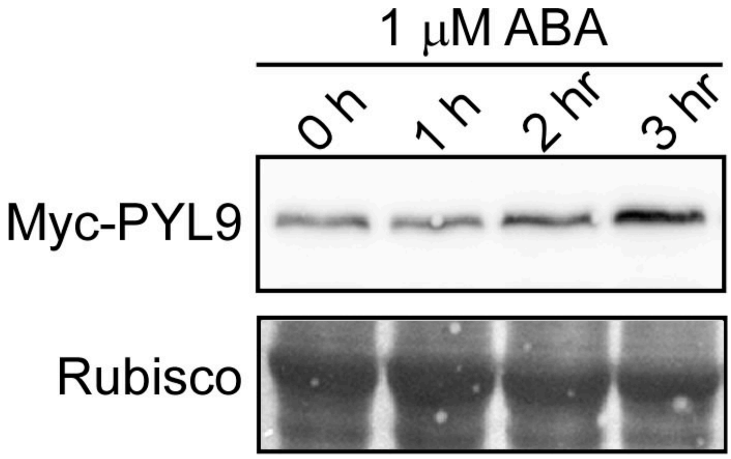

Supplementary Figure S5. Accumulation of the Myc-PYL9 protein induced by ABA. The transgenic plants containing *Myc-PYL9* in Col-0 background were treated by 1 μM ABA and the samples were harvested at the indicated time points. Coomassie brilliant blue (CBB) staining of Rubisco was used as loading control.

Supplementary Table 1. Primers used for plasmids construction.

Plasmids	Forward primer (5'-3')	Reverse primer (5'-3')
pCAMBIA1307-Myc-PYL9	ACGCGTCGACATGATGGACGGCGTTGAAGGC	GGGGTACCTCACTGAGTAATGTCCTGAG
pCAMBIA1307-FLAG-PUB22	ACGCGTCGACATGATGGATCAAGAGATAGAGATTC	GGGGTACCTCAAGCAGGATACGAATCATAC
BD-PYR1	CATGGAGGCCGAATTCATGCCTTCGGAGTTAACACC	GCAGGTCGACGGATCCTCACGTCACCTGAGAACCAC
BD-PYL1	CATGGAGGCCGAATTCATGGCGAATTCAGAGTCCTCC	GCAGGTCGACGGATCCTTACCTAACCTGAGAAGAGTTG
BD-PYL2	CATGGAGGCCGAATTCATGAGCTCATCCCCGGCCGTG	GCAGGTCGACGGATCCTTATTCATCATCATGCATAGGT
BD-PYL3	CATGGAGGCCGAATTCATGAATCTTGCTCCAATCCATG	GCAGGTCGACGGATCCTCAGGTCGGAGAAGCCGTG
BD-PYL4	CATGGAGGCCGAATTCATGCTTGCCGTTCACCGTCC	GCAGGTCGACGGATCCTCACAGAGACATCTTCTTCTTG
BD-PYL5	ACGAGGTCGGAATTCTCGCTGAGG	CGCGTCGACATAACTAATCATCAATTTGCC
BD-PYL6	CATGGAGGCCGAATTCATGCCAACGTCGATACAGTTTC	GCAGGTCGACGGATCCTTACGAGAATTTAGAAGTGTTCTC
BD-PYL7	CATGGAGGCCGAATTCATGGAGATGATCGGAGGAGAC	GCAGGTCGACGGATCCTCAAAGGTTGGTTTCTGTATG
BD-PYL8	CATGGAGGCCGAATTCATGGAAGCTAACGGGATTGAG	GCAGGTCGACGGATCCTTAGACTCTCGATTCTGTCGTG
BD-PYL9	CATGGAGGCCGAATTCATGGACGGCGTTGAAGGC	GCAGGTCGACGGATCC TCACTGAGTAATGTCCTGAG
BD-PYL10	CATGGAGGCCGAATTCATGAACGGTGACGAAACAAAGAAG	GCAGGTCGACGGATCCTCATATCTTCTTCTCCATAGATTC
BD-PYL13	CATGGAGGCCGAATTCATGGAAAGTTCTAAGCAAAAACG	GCAGGTCGACGGATCCTTACTTCATCATTTTCTTTGTGAGC
AD-PUB18	GGAGGCCAGTGAATTCAGTCATAGCAGCATGATCCATACG	CGAGCTCGATGGATCCCCCGAGCTAAATATACAAACA

AD-PUB22	GGAGGCCAGTGAATTCATGGATCAAGAGATAGAGATT	CGAGCTCGATGGATCCTCAAGCAGGATACGAATCATAC
AD-PUB23	GGAGGCCAGTGAATTCATGTCCGGAGGAATAATGGATG	CGAGCTCGATGGATCCTCAGCAGGGATATGCAAGAATC
pCold-GST-PYL5	CGAGGGATCCGAATTCATGAGGTCACCGGTGCAACTC	TAGACTGCAGGTCGACTTATTGCCGGTTGGTACTTCG
pCold-GST-PYL7	CGAGGGATCCGAATTCATGGAGATGATCGGAGGAGAC	TAGACTGCAGGTCGACTCAAAGGTTGGTTTCTGTATG
pCold-GST-PYL8	CGAGGGATCCGAATTCATGGAAGCTAACGGGATTGAG	TAGACTGCAGGTCGACTTAGACTCTCGATTCTGTCGTG
pCold-GST-PYL9	CGAGGGATCCGAATTCATGATGGACGGCGTTGAAGGC	TAGACTGCAGGTCGACTCACTGAGTAATGTCCTGAG
pCold-GST-PYL10	CGAGGGATCCGAATTCATGAACGGTGACGAAACAAAGAAG	TAGACTGCAGGTCGACTCATATCTTCTTCTCCATAGATTC
pCold-MBP-PUB22	CGAGGGATCCGAATTCATGGATCAAGAGATAGAGATTC	TAGACTGCAGGTCGACTCAAGCAGGATACGAATCATAC
pCold-MBP-PUB22C13A	CTTCCTTCTTCCTTGCTCCAATCTCTCTAG	CTAGAGAGATTGGAGCAAGGAAGAAGGAAG
pCold-MBP-PUB23	CGAGGGATCCGAATTCATGTCCGGAGGAATAATGGATG	TAGACTGCAGGTCGACTCAGCAGGGATATGCAAGAATC
pCold-MBP-PUB23C18A	CCTCCGTTCTTCCTTGCTCCTATCTCTTTGG	CCAAAGAGATAGGAGCAAGGAAGAACGGAGG
pCAMBIA1300-PUB22C13A-NUC	AGCTCGAGTAGTCGACATGGATCAAGAGATAGAG	ACGAGATCTGGTCGACAGCAGGATACGAATCATAC
pCAMBIA1300-CLUC-PYL9	ACGCGTCCCGGGGCGGTACCATGATGGACGGCGTTGAAGGC	GCCCTCTAGAGGATCCTCACTGAGTAATGTCCTGAG
pCAMBIA1300-CLUC-OsNAC2	TCCCGGGGCGGTACCATGGACTTGCCCCCTGGC	GCTCTGCAGGTCGACTTAGTAGCCCCATAGCGC
pSPYCE (M)-PUB22C13A	CGCCACTAGTGGATCCATCAAGAGATAGAGATT	TACCCTCGAGGTCGACAGCAGGATACGAATCATAC
pSPYNE173-PYL9	GCCTACTAGTGGATCCATGATGGACGGCGTTGAAGGC	TACCCTCGAGGTCGACTCACTGAGTAATGTCCTGAG

Supplementary Table 2. Primers used for the quantitative real-time PCR.

Plasmids	Forward primer (5'-3')	Reverse primer (5'-3')
ACTIN	ACTCTCCCGCTATGTATGTC	GATGGAAGAGCTGGTCTTTG
PYL9	AGCTCCTCTTCATCTCGTTTGG	AGACTGCCGATTTCAGGATCAC
Myc-PYL9	ATGGAGAGCTTGGGCGACCTCAC	ATCACCATCGTTCCTGCTCTTC
PUB22	TTCGGTTGGGAGGTTCTG	CAAACCCTAGCGTGAAGC
PUB23	GGCCGTGGAAGCTGGAGTAATC	CCCTAACCGCTCTATCGCTTGC
PUB24	TAGAGCCGAGATTCTTGC	ACCGTCCCAACATTAACC
NLUC	TTCTATCCGCTGGAAGATGGAACC	TTCATAGCTTCTGCCAACCGAACG
CLUC	TGGATGGCTACATTCTGGAGAC	GGTGTTGGAGCAAGATGGATTC
